# Prognostic Significance of Tumor Location for Liver Cancer Radiotherapy

**DOI:** 10.7759/cureus.3714

**Published:** 2018-12-11

**Authors:** A. Rashid Dar, Isabelle McKillop, Jason Vickress, Michael Lock, Slav Yartsev

**Affiliations:** 1 Radiation Oncology, Schulich School of Medicine & Dentistry - Western University, London, CAN; 2 Medical Physics, London Regional Cancer Program, London, USA; 3 Medical Physics, Peel Regional Cancer Center, Brampton, CAN; 4 Medical Physics, London Regional Cancer Program, London, CAN

**Keywords:** hepatic cancer, radiation therapy, treatment outcome prognosis

## Abstract

Introduction

According to the Surveillance, Epidemiology and End Results (SEER) data, cancerous involvement of the liver is on an increase over the last three decades. It occurs worldwide in all races and carries a poor prognosis. Currently, considerable progress has been made in patient selection, staging, surgery, chemotherapy agents, and stereotactic radiotherapy in both primary and metastatic liver cancers with improved outcomes. While there is evidence of the prognostic factors of liver function, the involvement of the portal vein, inferior vena cava thrombosis, lesion size, radiation dose, number of fractions, and SBRT techniques, there is no study evaluating outcomes with the location of the lesion. Our aim in this retrospective study was to explore the correlation of tumor location from the portal vein bifurcation (vascular wall) and the radiotherapy outcome (survival) in hepatocellular cancer.

Methods

Contrast-enhanced computed tomography (CT) studies in 86 patients with liver cancer were retrospectively reviewed in an institutional review board (IRB)-approved database to determine the distance to the bifurcation point of the portal vein from tumor’s centre of mass (distance tumor bifurcation: DTB) and from the edge point of the planning target volume closest to the bifurcation (distance edge bifurcation: DEB). The mean dose to the sphere of 1 cm diameter around the bifurcation point (mean dose at bifurcation: MDB) was calculated. These parameters were tested as predictors of patient outcomes using univariate and multivariate analysis as two groups of patients.

Results

Only the DEB correlation with survival for hepatocellular carcinoma (HCC) was found to be significant (P = 0.028). A larger MDB is caused by a smaller DTB and a smaller DEB. The hazard ratio for DTB, DEB, and MDB were 0.48, 0.41, and 1.05, respectively. The DEB was found to be a better predictor of outcomes (overall survival) compared to the DTB and MDB parameters. The close proximity of the tumor to the blood supply vessels was a decisive factor. The DTB parameter is also dependent on the size of the tumor and this factor weakens the correlation of this parameter on survival data. The inclusion of the dosimetric and geometric location, as well as distance parameters in predictive models for liver cancer patients, was shown to benefit the pre-selection of treatment options for liver cancer patients treated with radiotherapy.

Conclusion

For hepatocellular cancer patients, the distance between the edge point of the planning treatment volume (PTV) to the portal vein bifurcation (DEB) of more than 2 cm was found to be a predictor of survival.

## Introduction

Epidemiological studies report that liver cancer occurs in all races and is on an increase in many parts of the world, including North America, with a five-year overall survival rate of 19% [1-3]. There were approximately 854,000 incident cases of liver cancer and 810,000 deaths globally in 2015 [4]. The most common causes of liver cancer include the hepatitis B virus (HBV), hepatitis C virus (HCV), infection, and alcohol use. Liver cancer has rather limited treatment options; surgical resection remains the standard of care in selectively operable cases which form nearly 20%. Until recently, radiation played a palliative symptomatic control role because of the high radiosensitivity of liver cells with tolerance doses of 30 Gy in three to four weeks with conventional radiotherapy. Higher doses were considered too toxic and prohibitive.

Over the last two decades, advances in radiation oncology technology have allowed for improved sparing of healthy tissues, while delivering sufficiently high ablative doses to the tumor [5-11]. Vickress et al. have recently included radiation delivery planning parameters for analysis of the clinical outcomes in a large group of liver cancer patients with primary and metastatic disease [12]. In this paper, we investigate whether the location of the tumor with respect to the bifurcation of the blood vessels (portal vein) impacts the patient overall survival (OS), explore any correlation between the two, and propose the concept for future radiotherapy planning technique and research to improve treatment outcomes.

## Materials and methods

**Figure 1:**
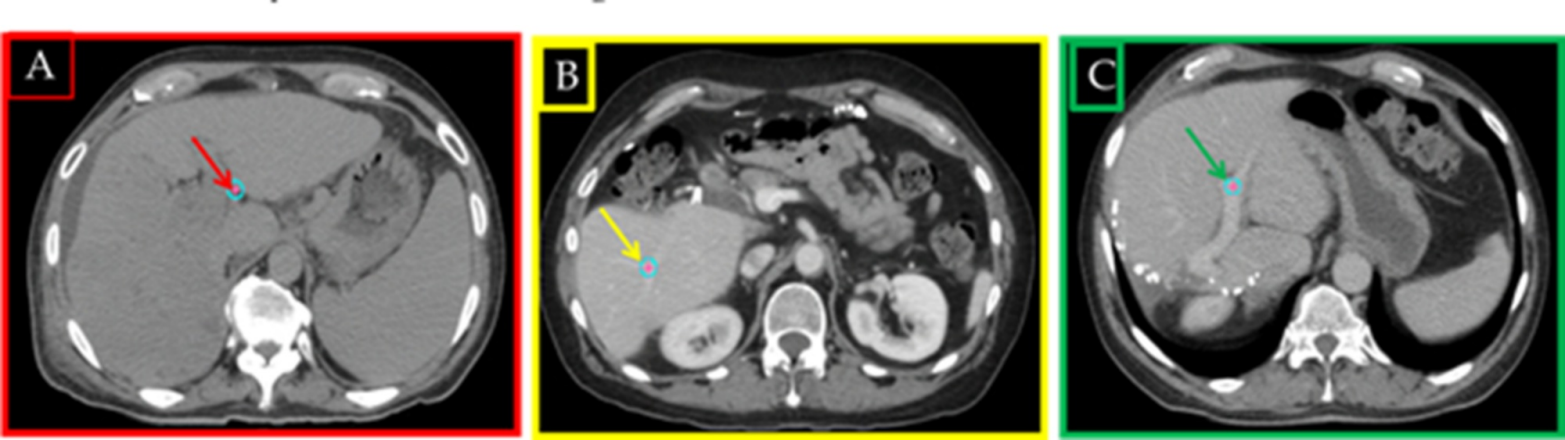
Bi-Point Location Accuracy on CT Scans A) not clear Bi-Point, determined with 1 cm accuracy; B) moderately clear Bi-Point, determined with 3 mm accuracy; C) crystal clear, unambiguous visualization of Bi-Point Bi-Point: bifurcation point; CT: computed tomography

An ethics board-approved database was retrospectively reviewed from 2011 to 2015. All selected 86 patients (51 males, 35 females) had a biopsy-proven liver cancer and dynamic contrast computed tomography (CT) studies obtained on the Philips Big Boar scanner (Philips Medical Systems, Cleveland, OH, USA) with respiratory gating. Radiotherapy planning with the Pinnacle TPS (Philips Medical Systems, Cleveland, OH, USA) was done for stereotactic body radiation therapy (SBRT) on the Varian accelerator (Varian Medical Systems, Palo Alto, CA, USA) with the volumetric modulated arc therapy (VMAT) technique and image-guided radiotherapy (IGRT) using cone beam CT (CBCT) technology. Contouring was done according to the Radiation Therapy Oncology Group (RTOG) criteria [13] with necessary standard gross tumor volume (GTV), planning target volume (PTV), normal liver, whole liver, portal vein, blood vessels, and other critical structures. The contours were reviewed again in detail by another physician for the quality assurance (QA) process. Overall survival (OS) and disease-free survival (DFS) data for all 86 patients were collected as per the Kaplan Meier estimator from follow-up notes. CT studies with and without contrast were analyzed in detail with MIM Vista fusion software, version 6.5 (MIM Software Inc., Cleveland, OH, USA) to determine the following three characteristics: 1) bifurcation point (Bi-Point): where the portal vein first splits, 2) target point: PTV center of the mass, and 3) edge point: point closest to the Bi-Point on the PTV contour. These data were used to calculate the distance from the target point to the Bi-Point (DTB) and from the edge point to the B-Point (DEB). The mean dose within the sphere of 1 cm diameter around the Bi-Point (MDB) was calculated. The clarity of Bi-Points on the CT scans was examined to determine the point location accuracy. Figure 1 represents the difference in a CT scan with A: not clear Bi-Point, determined with 1 cm accuracy, B: moderately clear Bi-Point, determined with 3 mm accuracy, and C: crystal clear, unambiguous visualization of the Bi-Point. The number of Bi-Points found within the PTV (16) and the gender of the patients was recorded. Seventy-four patients had one tumor, nine patients had two tumors, and three patients had ≥ 3 tumors as separate lesions, intrahepatic multifocal lesions, or intrahepatic oligometastatic disease. These 12 patients were also included in the study. The MIM Maestro, version 6.5 software (MIM Software Inc., Cleveland, OH, USA) was used to determine any correlation between the DEB, DTB, and MDB parameters and the overall survival (OS) data for these patients. Kaplan-Meier’s log graphs were created for OS comparison and statistical evaluation.  

## Results

**Figure 2:**
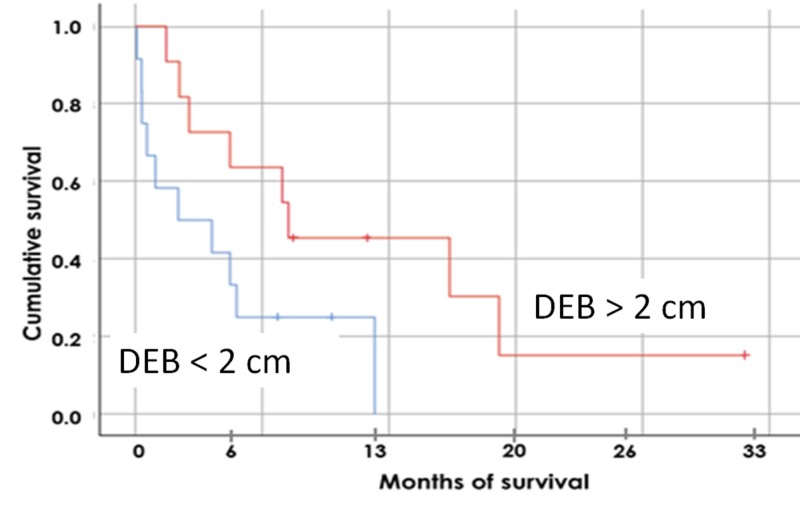
Survival of Patients with Primary HCC Versus DEB Bi-Point: bifurcation point; DEB: distance edge bifurcation; HCC: hepatocellular carcinoma

Demographic data are shown in Table 1. The DEB parameter correlation with OS was significant for only HCC patients with P = 0.028 (Table 1). For all patients, the MDB correlation was not significant. The DTB parameter was almost significant with P = 0.079. For patients with more than one lesion, the DEB was not significant. For all patients, the bilirubin, serum albumin, GTV, and an equivalent dose in 2-Gy fraction (EQD2Gy) were significant with a P-value < 0.05. When the DEB increases by 10 mm, the MDB decreases by approximately 3 Gy (y = -0.29 x + 22.24). When the DTB increases by 15 mm, the MDB decreases by approximately 3.5 Gy (y = -0.22 x + 27.54). When the DEB increases by approximately 6.5 mm, the DTB increases by 10 mm (y = 0.76 x - 18.05). The survival curve in Figure 2 indicates that the HCC patients, whose DEB was more than 2 cm, lived longer than those whose DEB was less than 2 cm.

**Table 1:** Correlation Between Different Patient Factors and Overall Survival for 23 HCC Patients Beta: regression coefficient; CI: confidence interval; DEB: distance edge bifurcation; DTB: distance tumor bifurcation; EQD2GY: equivalent total dose in 2-Gy fraction; GTV: gross tumor volume; HCC: hepatocellular carcinoma; HR: hazard ratio; MDB: mean dose at bifurcation; Q1: first quartile; Q3: third quartile

Factor	Beta	p-value	Q1	Q3	HR	95% CI
DEB	-0.033	0.028	10.85	38.08	0.41	0.18 - 0.91
DTB	-0.019	0.079	47.75	85.9	0.48	0.22 - 1.09
MDB	0.003	0.886	0.39	18.55	1.05	0.55 - 2
GTV	0.001	< 0.001	90.45	682.21	1.53	1.29 - 1.82
Bilirubin	0.024	< 0.001	9.15	22.55	1.37	1.19 - 1.58
Serum aIbumin	-0.091	< 0.001	31	40.5	0.42	0.27 - 0.67
EQD2Gy	-0.028	< 0.001	39.39	70	0.42	0.28 - 0.64

**Figure 3:**
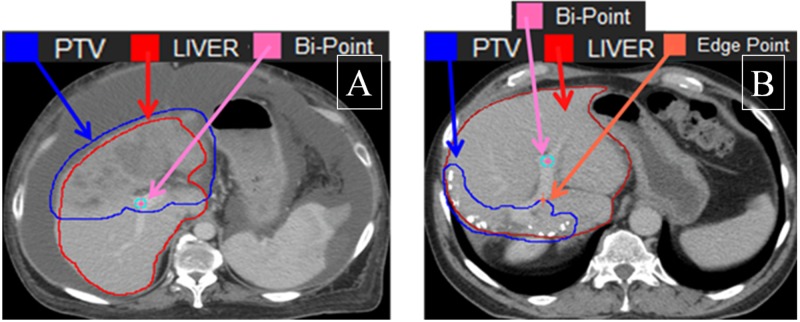
Correlation Between Distance Edge Bifurcation (DEB) and Mean Dose at Bifurcation (MDB) Example of positions of the Bi-Points relative to the PTV: A) Bi-Point within the PTV (16 cases); B) Bi-Point outside the PTV (70 cases). Bi-Point: bifurcation point; PTV: planning target volume

**Figure 4:**
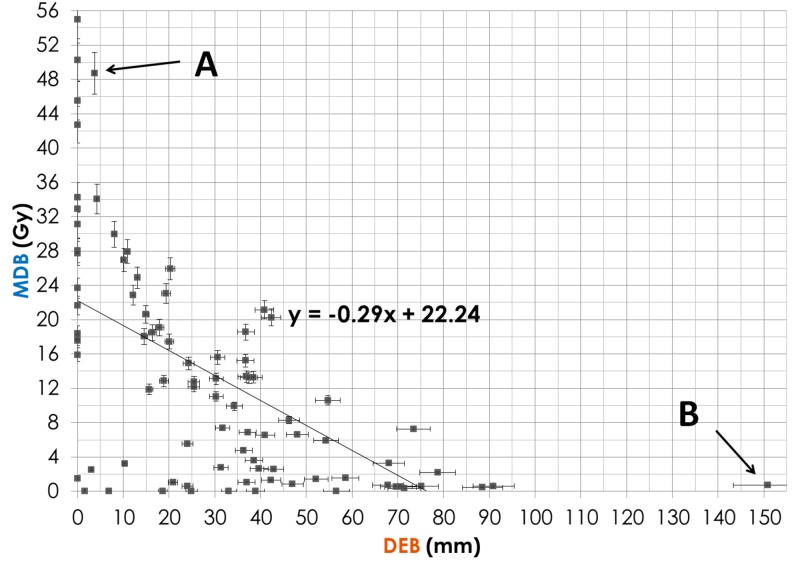
Correlation Between Distance Edge Bifurcation (DEB) and Mean Dose at Bifurcation (MDB) Point A is an outlier from the trend line and represents a patient whose Bi-Point was located only 3.7 mm from the edge point, thus making the DEB very low and the MDB extremely high because of the amount of radiation that would be exposed to the Bi-Point. Point B represents a patient whose tumor was located in the right posterior section of the liver, while the Bi-Point was in the bottom center region, thus making the dosage exposed to the Bi-Point much lower than for the other patients.

**Figure 5:**
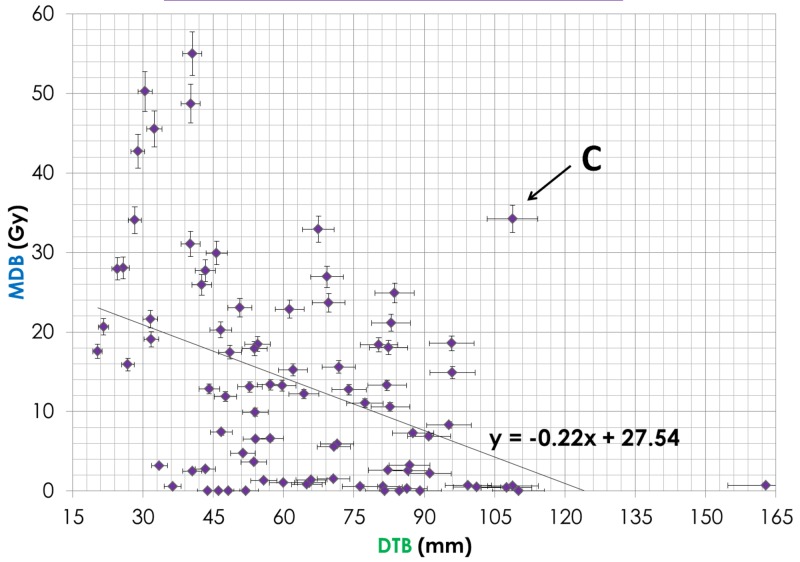
Correlation Between Distance Tumor Bifurcation (DTB) and Mean Dose at Bifurcation (MDB) Point C represents a patient whose DTB and MDB are both large because of the large tumor volume. When the tumor is large, the MDB is large because the radiation fields are wide and deposit more doses to healthy liver cells.

**Figure 6:**
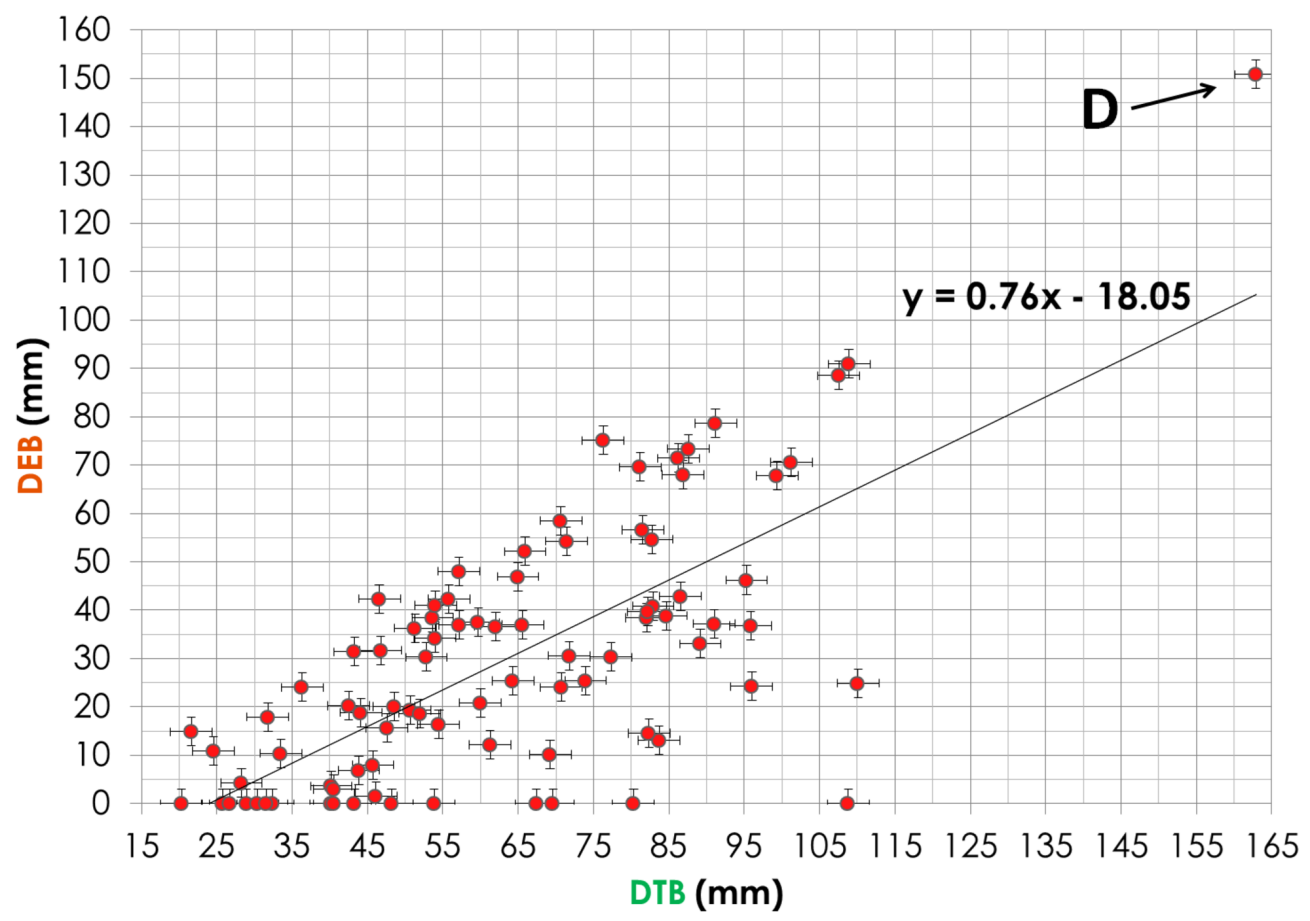
Correlation Between Distance Tumor Bifurcation (DTB) and Distance Edge Bifurcation (DEB) Point D corresponds to the patient’s tumor located at the bottom center region, thus making both DTB and DEB larger due to the small geometric size of the tumor.

For 16 patients, the Bi-Point was located within the PTV as demonstrated in Figure 3, resulting in a larger MDB, absence of the edge point (DEB = 0), and a small DTB. Some of these points are shown in Figure 4 where the DEB equaled zero, leading to a very high MDB. In the case of 12 patients who had more than one tumor, we used the tumor closest to the Bi-Point for calculation of the DEB and DTB. Point A in Figure 4 is an outlier from the trend line; this point represents a patient whose Bi-Point was located only 3.7 mm from the edge point, thus making the DEB very low and the MDB extremely high because of the amount of radiation that would be exposed to the Bi-Point. Point B on Figure 4 represents a patient whose tumor was located in the right posterior section of the liver, while the Bi-Point was in the bottom center region, thus making the dosage exposed to the Bi-Point much lower than for the other patients. In Figure 5, Point C represents a patient whose DTB and MDB are both large because of the large tumor volume. When the tumor is large, the MDB is large because the radiation fields are wide and deposit more doses to healthy liver cells. Point D in Figure 6 corresponds to the patient’s tumor located at the bottom center region, thus making both DTB and DEB larger due to the small geometric size of the tumor.

## Discussion

The liver is a unique, large, glandular organ supplied by the dual blood supply of the portal venous and hepatic arterial systems. Also, there is a biliary system of tributaries collecting and draining into the bile ducts [14]. These are architecturally intertwined with multiple functions, offering a site of various pathological processes which leave their mark as fibrosis. Also, the liver can be involved in benign adenomas and malignant neoplastic process, which, according to epidemiological studies, is on an increase in liver cancer worldwide [2-3].

These structural and functional aspects are important considerations in any therapeutic surgical or radiation therapeutic interventions for outcomes and toxicities locoregionally. Also, the local spread pattern and microvascular invasion impact outcome [15].

The bifurcation of the major vessel portal vein inside the liver was taken as a landmark for our study for evaluating the distance DEB to the PTV as a practical tool for radiation planning parameters and outcomes. Other potential evaluating metrics, DTB and MDB, correlate with the DTB parameter, as shown in Figures 4-6, but their correlation with the survival data was not as strong. It is possible that hypoxia and lesion size are related to the proximity of the PTV to the portal vessel wall and this may cause a poor outcome for these patients as per Figure 2. Our hypothesis is that, for a large tumor mass, the burden of cancerous cells is close to the portal vein branch, providing easy access to vascular spread within the liver. Larger tumors have a greater hypoxic component leading to radioresistance, possibly even in the case of the SBRT dose as shown recently by Kelada et al. [16].

Although our tool is based on contrast-enhanced CT studies, we feel it is easily reproducible in other modes of imaging, thus allowing inter-comparisons of various interventions, response assessment, and radiogenomic studies. Our tool can be also helpful for the prognostication of target agents, such as sorafenib or newer agents approved as first-line treatments, and for possible surgical or high-dose ablation therapy or transition to transplant or re-treatment.

With the abundance of literature on clinical prognostic factors, such as size and number of lesions, total radiation dose, fractionation, biological effective dose, techniques, patient factors, performance status, Child-Pugh classification, vascular invasion, thrombosis, and prior treatments, there are no pre-treatment radiotherapy planning criteria with respect to tumor location correlated with survival [9, 17]. We believe this will help the Barcelona Classification of Treatment Guide recently published by the National Cancer Institute, United States (NCI US) [2]. To our knowledge, this is the first attempt to guide radiation planning and research based on the geometric location of the tumor and assessment of the constraints within the liver.

Our concept of the DEB parameter as a tool in treatment choice and prognosis needs further evaluation by a large dataset. A limitation of our study was an insufficient number of patients for fitting to several prognostic groups. Also, there were few patients (12) with ≥ 1 lesion; however, the concept of DEB still holds for these cases. Although a large number of patients studied helped to develop a hypothesis, future validation with an independent cohort of patients is necessary. The study has a limitation of a retrospective character; however, a significant number of patients were analyzed to draw our hypothesis. 

## Conclusions

The dose to the bifurcation point is larger for the patients where tumors are further away (larger DEB parameter). For patients with HCC whose DEB is more than 2 cm from the bifurcation of the portal vein, the probability of a better outcome is far greater.

## References

[1] Høyer M, Swaminath A, Bydder S (2012). Radiotherapy for liver metastases: a review of evidence. Int J Radiat Oncol Biol Phys.

[2] Ohri N, Dawson LA, Krishnan S (2016). Radiotherapy for hepatocellular carcinoma: New indications and directions for future study. J Natl Cancer Inst.

[3] Islami F, Miller KD, Siegel RL (2017). Disparities in liver cancer occurrence in the United States by race/ethnicity and state. CA Cancer J Clin.

[4] Global Burden of Disease Liver Cancer Collaboration (2017). The burden of primary liver cancer and underlying etiologies from 1990 to 2015 at the global, regional, and national level: results from the Global Burden of Disease Study 2015. JAMA Oncol.

[5] Lo CH, Yang JF, Liu MY (2017). Survival and prognostic factors for patients with advanced hepatocellular carcinoma after stereotactic ablative radiotherapy. PLoS One.

[6] Kress MA, Collins BT, Collins SP (2012). Scoring system predictive of survival for patients undergoing stereotactic body radiation therapy for liver tumors. Radiat Oncol.

[7] Park SH, Kim JC, Kang MK (2016). Technical advances in external radiotherapy for hepatocellular carcinoma. World J Gastroenterol.

[8] Crane CH, Koay EJ (2016). Solutions that enable ablative radiotherapy for large liver tumors: fractionated dose painting, simultaneous integrated protection, motion management, and computed tomography image guidance. Cancer.

[9] Doi H, Beppu N, Kitajima K, Kuribayashi K (2018). Stereotactic body radiation therapy for liver tumors: current status and perspectives. Anticancer Res.

[10] Meyer J, Singal AG (2018). Stereotactic ablative radiotherapy for hepatocellular carcinoma: history, current status, and opportunities. Liver Transpl.

[11] Murray LJ, Dawson LA (2007). Advances in stereotactic body radiation therapy for hepatocellular carcinoma. Semin Radiat Oncol.

[12] Vickress J, Lock M, Lo S (2017). A multivariable model to predict survival for patients with hepatic carcinoma or liver metastasis receiving radiotherapy. Future Oncol.

[13] Hong TS, Bosch WR, Krishnan S (2014). Interobserver variability in target definition for hepatocellular carcinoma with and without portal vein thrombus: radiation therapy oncology group consensus guidelines. Int J Radiat Oncol Biol Phys.

[14] Juza RM, Pauli EM (2014). Clinical and surgical anatomy of the liver: a review for clinicians. Clin Anat.

[15] Rodríguez-Perálvarez M, Luong TV, Andreana L (2013). A systematic review of microvascular invasion in hepatocellular carcinoma: diagnostic and prognostic variability. Ann Surg Oncol.

[16] Kelada OJ, Decker RH, Nath SK (2018). High single doses of radiation may induce elevated levels of hypoxia in early-stage non-small cell lung cancer tumors. Int J Radiat Oncol Biol Phys.

[17] Tanguturi SK, Wo JY, Zhu AX (2014). Radiation therapy for liver tumors: ready for inclusion in guidelines?. Oncologist.

